# Prognostic value and immunological role of BAIAP2L2 in liver hepatocellular carcinoma: A pan-cancer analysis

**DOI:** 10.3389/fsurg.2022.985034

**Published:** 2022-10-21

**Authors:** Xiudan Han, Wei Long, Ying Liu, Jixiong Xu

**Affiliations:** ^1^ Department of Endocrinology and Metabolism, First Affiliated Hospital of Nanchang University, Nanchang, China; ^2^ Jiangxi Clinical Research Center for Endocrine and Metabolic Disease, Nanchang, China; ^3^ Jiangxi Branch of National Clinical Research Center for Metabolic Disease, Nanchang, China; ^4^ Department of Rheumatology, First Affiliated Hospital of Nanchang University, Nanchang, China

**Keywords:** BAIAP2L2, liver hepatocellular carcinoma (LIHC), prognostic value, immune infiltration, pan-cancer analysis

## Abstract

**Background:**

In recent years, the role of BAI1-associated protein 2-like 2 (BAIAP2L2) in the prognosis and immune microenvironment of various cancers has attracted increasing attention. However, its clinical value and immune infiltration in liver hepatocellular carcinoma (LIHC) remain unclear.

**Objective:**

To investigate the prognostic value of BAIAP2L2 and its correlation with immune infiltration in LIHC, we conducted corresponding data mining.

**Methods:**

In this study, The Cancer Genome Atlas, GTEx, StarBase, UALCAN, TIMER, GEPIA, Human Protein Atlas, Kaplan–Meier Plotter, cBioPortal, LinkedOmics, STRING and BioGPS databases were used to analyze BAIAP2L2 in cancers. Logistic regression and Cox regression were performed to analyze the correlation between clinical features and BAIAP2L2 expression in LIHC. In addition, the diagnostic and prognostic values of BAIAP2L2 in LIHC were determined by receiver operating characteristic (ROC) curves and nomograms. Single-sample gene set enrichment analysis (ssGSEA), BioGPS and TIMER were used to analyze the correlation between BAIAP2L2 and immune infiltration. More importantly, quantitative real-time polymerase chain reaction was used to verify BAIAP2L2 expression in a liver cancer cell line and a normal cell line. Visualization of data was mostly achieved using R language, version 3.6.3.

**Results:**

High BAIAP2L2 levels indicated poor overall survival (OS) and disease-free survival (DFS) of patients with LIHC. Abnormally increased expression of BAIAP2L2 in LIHC may be the result of both genetic alterations and lower DNA methylation levels. Furthermore, Cox regression analysis showed that high BAIAP2L2 expression was an independent risk factor for OS and DFS in patients with liver cancer. ROC curves and nomograms also confirmed the diagnostic and prognostic values of BAIAP2L2 in LIHC. Additionally, a PPI network of BAIAP2L2 was established and results implyed that BAIAP2L2 interacts with MTSS1, AMPH, FCHO1, SYT9, PDK2, MTSS1L, PM20D1, CHST4 and PALM3. ssGSEA showed that BAIAP2L2 was associated with T cells and natural killer cells. Simultaneously, the TIMER database showed that the expression of BAIAP2L2 in LIHC was positively correlated with tumor infiltrating cells, including B cells, CD8+ T cells, CD4+ T cells, macrophages, neutrophils and dendritic cells.

**Conclusions:**

Through pan-cancer analysis, prognostic and immunological value of BAIAP2L2 in LIHC was identified. This is the first report on the potential of BAIAP2L2 as a prognostic biomarker and its correlation with immune infiltration in LIHC.

## Introduction

Due to the increasing prevalence of established risk factors, such as the growth and aging of the population, smoking, being overweight, lacking physical activity, urbanization and changes in reproductive patterns resulting from economic development, the incidence of cancer is gradually rising and has become a huge burden on society (1). Liver hepatocellular carcinoma (LIHC; also known as HCC) is the sixth most commonly diagnosed cancer and the fourth leading cause of cancer mortality in the world, with approximately 841,000 new cases and 782,000 deaths annually (2). In the past 30 years, the incidence of HCC has been on the rise worldwide. The World Health Organization estimates that more than 1 million patients will die from liver cancer in 2030 (3). The highest incidence rates of HCC were mainly observed in Northern and Western Africa (Egypt, the Gambia, Guinea) and Eastern and Southeastern Asia (Mongolia, Cambodia, and Vietnam) (2). The dominant risk factors for HCC are chronic infection with hepatitis B virus or hepatitis C virus, nonalcoholic fatty liver disease, alcohol consumption, cigarette smoking and environmental toxins (4). The treatment of HCC mainly includes surgical resection, liver transplantation, vascular intervention and radiofrequency ablation (5). Despite the emergence of targeted therapy, advanced-stage LIHC remains largely incurable due to low response rate and therapeutic resistance (6). Moreover, most of tumours response to immunotherapies is either non-existent or short-lived (7). To improve the survival rate of LIHC patients, new therapeutic targets will still need to be discovered so that existing drug options can be increased and a better understanding of the underlying mechanisms leading to drug resistance can be gained.

BAIAP2L2 (BAI1-associated protein 2-like 2; also known as Pinkbar) is located on chromosome 22q131 (8). Along with BAIAP2L1, IRSp53, MTSS1 and MTSS1L, BAIAP2L2 belongs to the I-BAR (Inverse Bin-Amphiphysin-Rvs) subfamily (9, 10). They have different isoforms, but all contain an N-terminal I-BAR domain. In recent years, the I-BAR family has been found to be related to the occurrence of tumors (11, 12). Overexpression of IRTKs was negatively correlated with overall survival in patients with gastric cancer (13). BAIAP2L1 is also a potential biomarker in ovarian cancer (14) and IRSP53 plays an important role in regulating the motility/invasion of cancer cells (15, 16). Studies have shown that MTSS1 is notably downregulated during the progression of gastric cancer (17), and hypermethylated MTSS1 can promote the migration of prostate cancer (18). The key role of I-BAR family members in the carcinogenesis process. Although BAIAP2L2 was associated with the development of various cancers, including osteosarcoma (19), gastric cancer (20), Prostate Cancer (21) and lung cancer (22). However, no studies have reported the relationship between BAIAP2L2 and liver cancer.

In this study, we comprehensively analyzed the expression profile and prognostic value of BAIAP2L2 in 33 types of cancer, and found that BAIAP2L2 was highly expressed in LIHC. Overexpression of BAIAP2L2 is associated with poor prognosis of LIHC. Results also illustrated the immunological role of BAIAP2L2 in LIHC.

## Materials and methods

### Gene expression analysis

The “Diff Exp” module in the TIMER database (https://cistrome.shinyapps.io/timer/) allows us to study the differential expression of BAIAP2L2 in The Cancer Genome Atlas (TCGA) between tumor and adjacent normal tissues (23, 24). Distributions of gene expression levels are displayed using box plots, with statistical significance of differential expression evaluated using the Wilcoxon test. The GTEx database contains data for normal tissue. If the normal sample size of TCGA is insufficient, we will combine GTEx and TCGA to analyze the differential expression of BAIAP2L2 in tumors. Additionally, StarBase (http://starbase.sysu.edu.cn/) (25), a comprehensive online tool, was also applied to analyze gene expression. The “ENCORI Pan-Cancer Analysis Platform” in the StarBase database is designed to analyze the gene expression profile of 32 cancer types. The expression data of cancers were downloaded from TCGA project *via* Genomic Data Commons Data Portal. Then, the Venn diagrams of data from three database were plotted using the “ggplot2” R package. Finally, the UALCAN database (http://ualcan.path.uab.edu/analysis.html) [26] and Human Protein Atlas (HPA) databases (https://www.proteinatlas.org/) (27) were used to verify the differentiation of BAIAP2L2 expression levels between tumors and normal tissues. Adjusted *p* < 0.05 and |log2fold change (FC)| > 1 were chosen as the cutoff criteria.

### Survival analysis

To explore the prognosis of BAIAP2L2 across cancers, we used the Kaplan–Meier Plotter database (https://kmplot.com/analysis/) (28) and LinkedOmics (http://www.linkedomics.org/) (29) to analyze the effect of BAIAP2L2 on the survival of various cancers. The data sources for the Kaplan–Meier Plotter database include not only the Gene Expression Omnibus (GEO) but also the European Genome-Phenome Archive (EGA) and TCGA. To analyze the prognostic value of a gene, the patient samples are split into two groups according to various quantile expressions. Then, the two patient cohorts are compared by a survival plot, and the hazard ratio with 95% confidence intervals (CI) and logrank *p*-value are calculated. 95% CIs and a *p* value <0.05 were considered statistically significant.

### CNA and DNA methylation alteration analysis

The cBioPortal for cancer genomics (https://www.cbioportal.org/) was used to query the BAIAP2L2 characteristics of genetic mutations (30, 31). The copy number alteration (CNA) data and mutation type were displayed in the “Cancer Types Summary” module of TCGA database. DNA methylation levels of the BAIAP2L2 promoter in normal and tumor tissues were analyzed using the UALCAN database (http://ualcan.path.uab.edu/index.html). TCGA-assembler pipeline was used to download TCGA DNA methylation data generated using the Illumina HumanMethylation450 BeadChip. Downloaded data were further processed to calculate an average methylation (beta) value for each gene, considering CpG sites located in the promoter region of the gene *via* the TCGA-assembler (32).

### BAIAP2L2 expression-correlated gene and protein analysis

We predicted the genes and proteins interacting with BAIAP2L2 using LinkedOmics and the STRING database (https://string-db.org/) (33), respectively. To further explore the biological functions of BAIAP2L2 in LIHC, Gene Set Enrichment Analysis (GSEA) was used to analyze the Gene Ontology (GO) terms and Kyoto Encyclopedia of Genes and Genomes (KEGG) pathways. GO analysis is a powerful bioinformatics tool used to identify biological processes (BPs), cellular components (CCs) and molecular functions (MFs).The main goal of the KEGG database project is to assign functional meaning to genes and genomes at the molecular and higher levels. A protein–protein interaction (PPI) network of BAIAP2L2 was generated using the STRING database.

### Immune infiltration analysis

Immune infiltration analysis of LIHC was performed using single-sample gene set enrichment analysis (ssGSEA) in the “GSVA” R package, and the infiltration levels of 24 types of immune cells were quantified from gene expression profiles. The TIMER database and GEPIA databases (http://gepia.cancer-pku.cn/) (34) were used to explore the correlation between BAIAP2L2 expression and immune infiltration. We utilized the “Gene” module to estimate the correlation between BAIAP2L2 expression and immune infiltration levels (B cells, CD4+ T cells, CD8+ T cells, neutrophils, macrophages and dendritic cells). Then, the “Correlation” module was applied to analyze the association between BAIAP2L2 and prognosis-related immune cell markers to further estimate the potential infiltrating immune cell subtypes. The correlation coefficient was determined by the Spearman method, and *p values* were corrected using the Benjamini-Hochberg method. Furthermore, BioGPS (http://biogps.org/) is a centralized gene annotation portal that enables researchers to access distributed gene annotation resources (35). We used this database to display the level of BAIAP2L2 mRNA expression in human tissues and immune cells.

### Cell culture and quantitative real-time polymerase chain reaction (qRT–PCR) of cell lines

The human hepatocarcinoma cell line HepG2 and human normal liver cell line LO2 were cultured in Minimum Essential Medium (MEM, Procell) and Roswell Park Memorial Institute 1640 (RPMI 1640, Procell), respectively, containing 10% fetal bovine serum (FBS, Excell Bio) and antibiotics (100 units/ml penicillin and 100 µg/ml streptomycin) at 37°C and 5% CO2 in an incubator. qRT–PCR was conducted to evaluate gene expression. Total RNA was extracted from cell lines with TRIzol reagent in accordance with the manufacturer's instructions. Using a reverse transcription kit, the RNA was reverse transcribed into cDNA, and qRT–PCR analyses were quantified with SYBR Green (VAZYME). BAIAP2L2 expression was calculated based on the 2−ΔΔCt method with actin as an internal reference. qRT–PCR was performed in triplicate using samples derived from three independent experiments. Primers for BAIAP2L2 (forward: 5′-AGTTCATCAAAGACAGCCGC-3′, reverse: 5′-CAGGTGCTTCTCTGCTAGGA-3′) and *β*-actin (forward: 5′-CACGATGGAGGGGCCGGACTCATC-3′, reverse: 5′-TAAAGACCTCTATGCCAACACAGT-3′) were used for qRT–PCR.

### Statistical analysis

Most of the statistical analyses were performed using the bioinformatic tools mentioned above. The results were shown as the mean ± SD. IBM SPSS statistics 26.0 software was utilized for statistical analysis. A *p* value <0.05 was considered statistically significant.

## Results

### BAIAP2L2 is universally over-expressed in human pan-cancer

**Figure 1:**
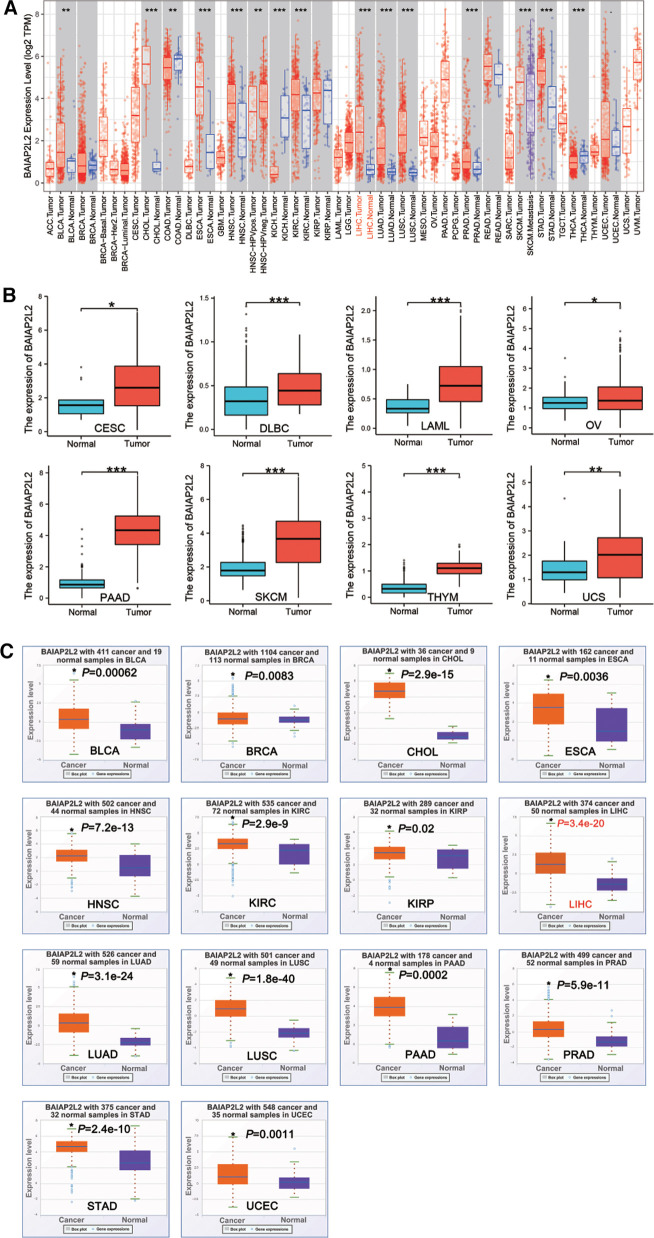
BAIAP2L2 expression levels in different types of cancer. (**A**) BAIAP2L2 expression levels in different types of cancer from TCGA datasets in TIMER. (**B**) BAIAP2L2 expression levels in different types of cancer from GTEX database combined with the TCGA database. *p < 0.05, **p < 0.01, ***p < 0.001. **(C**) Upregulated transcriptional level of BAIAP2L2 in pan-cancer samples from the StarBase database. The orange and purple boxes represent cancer and Normal samples, respectively, *p < 0.05 compared to the Normal tissue.

BAIAP2L2 has been proved to be abnormally expressed in cancers (36, 37). We used TIMER and StarBase database to demonstrate BAIAP2L2 expression in 33 types of human cancer. Data from the TIMER database showed that BAIAP2L2 expression was significantly increased in 10 types of cancer, including bladder urothelial carcinoma (BLCA), cholangiocarcinoma (CHOL), esophageal carcinoma (ESCA), head and neck squamous cell carcinoma (HNSC), kidney renal clear cell carcinoma (KIRC), LIHC, lung adenocarcinoma (LUAD), lung squamous cell carcinoma (LUSC), prostate adenocarcinoma (PRAD) and stomach adenocarcinoma (STAD) (Figure 1A, *p *< 0.05). Because some cancers in the TIMER database did not have normal tissue, we used the GTEX database combined with the TCGA database to supplement these data. The results showed that BAIAP2L2 was obviously increased in some cancers, including cervical squamous cell carcinoma and endocervical adenocarcinoma (CESC), lymphoid neoplasm diffuse large B-cell lymphoma (DLBC), acute myeloid leukemia (LAML), ovarian serous cystadenocarcinoma (OV), skin cutaneous melanoma (SKCM), pancreatic adenocarcinoma (PAAD), thymoma (THYM) and uterine carcinosarcoma (UCS) (Figure 1B, *p* < 0.05).

From StarBase database, we discovered that BAIAP2L2 was evidently upregulated in 14 types of cancer, including BLCA, breast invasive carcinoma (BRCA), CHOL, ESCA, HNSC, KIRC, kidney renal papillary cell carcinoma (KIRP), LIHC, LUAD, LUSC, PAAD, PRAD, STAD and uterine corpus endometrial carcinoma (UCEC) (Figure 1C, *p* < 0.05). By comparing results from the three databases (TIMER, GTEX and STARBASE), we concluded that BAIAP2L2 was generally overexpressed across 11 types of human cancer, including BLCA, CHOL, ESCA, HNSC, KIRC, LIHC, LUAD, LUSC, PAAD, PRAD and STAD (Figure 4A).

### BAIAP2L2 expression is closely associated with patient survival in human pan-cancer

**Figure 2:**
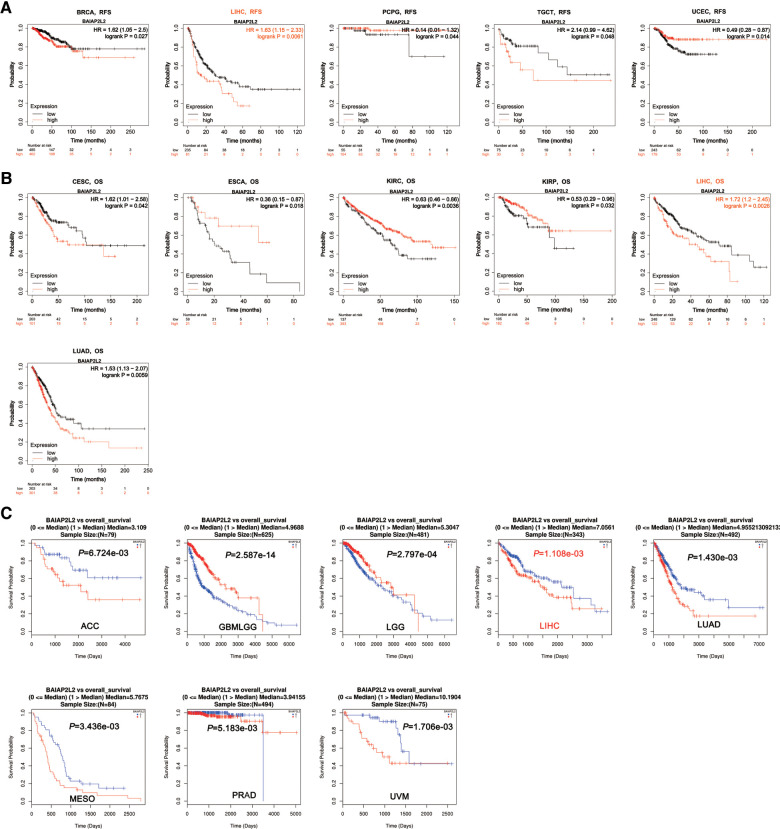
Survival analyses of BAIAP2L2 expression in different types of cancer. (**A**) Survival analysis of RFS in Kaplan–Meier plotter. (**B**) The survival analysis of OS in Kaplan–Meier plotter. (**C**) The survival analysis of OS From LinkedOmics.

Considering that BAIAP2L2 is dysregulated in a variety of cancers, we wanted to know whether its expression is related to the survival of cancer patients. Two databases, the Kaplan–Meier Plotter database and LinkedOmics, were utilized to analyze the relationship between BAIAP2L2 expression and patient overall survival (OS) in 33 types of cancer. The results from the Kaplan–Meier Plotter database demonstrated that a high level of BAIAP2L2 indicated unfavorable OS in CESC (*p *= 0.042), LIHC (*p *= 0.0026), and LUAD (*p *= 0.0059) and good OS in ESCA (*p *= 0.018), KIRC (*p *= 0.0036), and KIRP (*p *= 0.032) (Figure 2B). From LinkedOmics, we found that elevated BAIAP2L2 expression predicted worse OS in adrenocortical carcinoma (ACC) (*p *= 6.724e-03), LIHC (*p *= 1.108e-03), LUAD (*p *= 1.430e-03), mesothelioma (MESO) (*p *= 3.436e-03), PRAD (*p *= 5.183e-03), uveal melanoma (UVM) (*p *= 1.706e-03) and good OS in glioma (*p *= 2.587e-14) and brain lower grade glioma (LGG) (*p *= 2.797e-04) (Figure 2C).

Next, the Kaplan–Meier Plotter database was used to explore the relationship between BAIAP2L2 levels and patient disease-free survival (DFS) in 33 types of cancer. The results showed that a high level of BAIAP2L2 indicated poor DFS in BRCA (*p *= 0.027), LIHC (*p *= 0.0061), and testicular germ cell tumors (TGCTs) (*p *= 0.048) and good DFS in pheochromocytoma and paraganglioma (PCPG) (*p *= 0.044) and UCEC (*p *= 0.014) (Figure 2A). By comparing the results of Figures 1, 2, we found that high BAIAP2L2 levels were significantly correlated with poor patient OS and DFS in LIHC (Figures 4B,C). These data imply that BAIAP2L2 has potential prognostic value in LIHC.

### CNA and DNA methylation alterations of BAIAP2L2 across different human cancers

**Figure 3:**
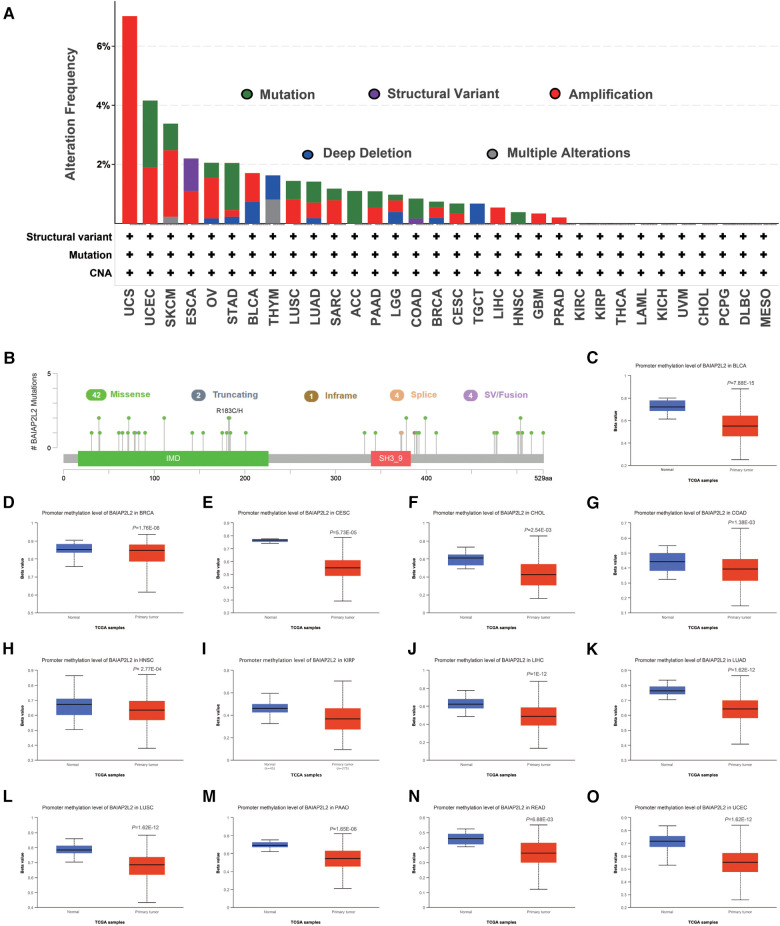
BAIAP2L2 mutated landscapes and methylation levels. (**A**) BAIAP2L2 mutation frequency in multiple TCGA pancancer studies according to the cBioPortal database. (**B**) Mutation diagram of BAIAP2L2 in different cancer types across protein domains. (**C–O**) BAIAP2L2 methylation levels were determined by UALCAN, and different beta value cutoffs have been considered to indicate hypermethylation [beta value: 0.7–0.5] or hypomethylation [beta value: 0.3–0.25].

Genetic and epigenetic changes play a significant role in regulating cancer development and immune tolerance (38). As shown in Figure 3A, we can see that elevated BAIAP2L2 expression was accompanied by gene alterations in BLCA, ESCA, LIHC, LUAD, LUSC, HNSC, PAAD, PRAD and STAD. It is worth noting that all LIHC cases with genetic alterations had amplification of BAIAP2L2. The types, sites and case number of the BAIAP2L2 genetic alteration are further presented in Figure 3B, and we found that missense mutation of BAIAP2L2 was the primary type of genetic alteration.

DNA methylation is an epigenetic mechanism that can affect the progression of tumors (39). To correlate promoter DNA methylation levels with BAIAP2L2 expression, we explored the differential promoter DNA methylation status of BAIAP2L2 between tumors and adjacent normal tissues by using UALCAN (Figures 3C–O). we found that BAIAP2L2 had lower DNA methylation levels in LIHC. Overall, abnormally increased expression of BAIAP2L2 mRNA in LIHC may be the result of both genetic alterations and lower DNA methylation levels.

### BAIAP2L2 is up-regulated and is associated with poor prognosis in LIHC

**Figure 4:**
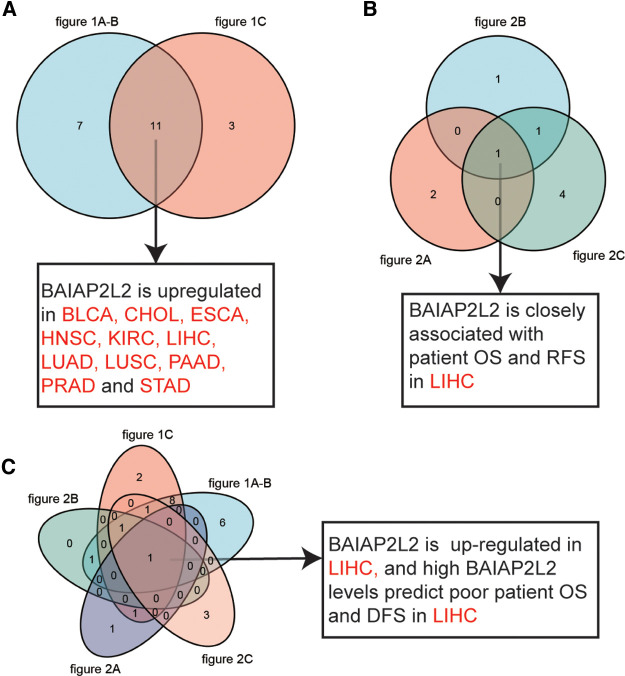
Data comparison is shown as Venn diagrams. (**A**) Comparative analysis of data in Figure 1, BAIAP2L2 is overexpressed in BLCA, CHOL, ESCA, HNSC, KIRC, LIHC, LUAD, LUSC, PAAD, PRAD and STAD. (**B**) BAIAP2L2 is closely associated with patient OS and RFS in LIHC (Figure 2). (**C**) High BAIAP2L2 levels predict poor patient OS and DFS in LIHC (Figures 1, 2).

By comprehensive analysis of the expression (Figure 1) and prognosis (Figure 2) of BAIAP2L2 across cancers, we found that BAIAP2L2 was overexpressed in LIHC and was closely associated with poor prognosis of liver cancer patients (Figure 4), implying its importance in predicting the clinical outcome of LIHC. Therefore, we focused on investigating the function of BAIAP2L2 in LIHC.

**Figure 5:**
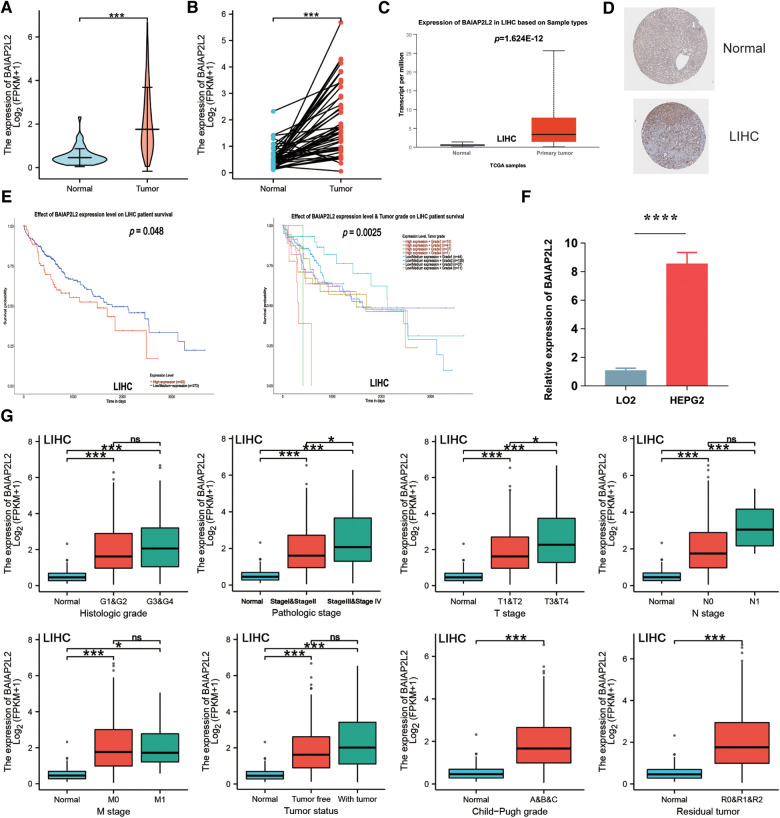
Validation of the expression level of BAIAP2L2 and high BAIAP2L2 expression is correlated with clinical characteristics in patients with LIHC. (**A**) The differential expression of BAIAP2L2 in LIHC samples and unpaired adjacent samples from TCGA. (**B**) The differential expression of BAIAP2L2 in 50 LIHC samples and matched adjacent samples from TCGA. (**C**) Validation of BAIAP2L2 at the mRNA level using the UALCAN database. (**D**) Validation of BAIAP2L2 at the protein level using the HPA database. (**E**) Survival analyses of BAIAP2L2 expression in LIHC. (**F**) Validation of BAIAP2L2 at the mRNA level *in vitro* using qRT–PCR analysis. Relative BAIAP2L2 mRNA levels in a LIHC cell line (HEPG2) and a human Normal liver cell line (LO2). (**G**) Box plot assessing BAIAP2L2 expression in patients with LIHC according to different clinical characteristics using the UALCAN database. *p < 0.05, **p < 0.01, ***p < 0.001, ****p < 0.0001.

Firstly, BAIAP2L2 expression in LIHC samples and adjacent normal tissues was analyzed through TCGA. BAIAP2L2 expression was observably elevated in LIHC tissues (Figures 5A,B). Then, the results from the UALCAN database showed that BAIAP2L2 was dramatically upregulated in LIHC compared to normal tissues (Figure 5C), and a high level of BAIAP2L2 indicated unfavorable survival probability in LIHC (Figure 5E). In addition, we examined the protein level of BAIAP2L2 in LIHC using HPA and discovered that BAIAP2L2 was overexpressed in liver cancer (Figure 5D). More importantly, qRT–PCR was conducted to evaluate gene expression, and we found that BAIAP2L2 mRNA expression was upregulated in a LIHC cell line (HEPG2) compared to a human normal liver cell line (LO2) (*p* < 0.0001) (Figure 5F).

### High BAIAP2L2 expression is correlated with clinical characteristics and pathological parameters in patients with LIHC

Clinical characteristics and gene expression data of 371 patients with LIHC were obtained from TCGA database. According to the mean value of BAIAP2L2, the patients with LIHC were divided into the high expression group and low expression group (Table 1), and then the Wilcoxon rank sum test and logistic regression were used to analyze the correlation between BAIAP2L2 expression and clinical features. High BAIAP2L2 expression was associated with histologic grade, pathologic stage, T stage, N stage, M stage, tumor status, Child-Pugh grade and residual tumor (Figure 5G). The results of univariate analysis using logistic regression demonstrated that BAIAP2L2 expression was connected with poor prognostic clinical characteristics in patients with LIHC (Table 2). High BAIAP2L2 expression was linked to sex [odds ratio (OR) = 2.149, 95% CI = 1.382–3.372, *p *< 0.001], age (OR = 0.662, 95% CI = 0.438–0.996, *p *= 0.048), histologic grade (G3/G4 vs. G1/G2: OR = 1.855, 95% CI = 1.208–2.867, *p* = 0.005) and AFP (OR = 2.544, 95% CI = 1.441–4.586, *p *= 0.002).

**Table 1:** Correlation between BAIAP2L2 expression and clinicopathologic characteristics of patients with LIHC.

Characteristic	Low expression of BAIAP2L2	High expression of BAIAP2L2	*p*
*n*	185	186	
Gender, *n* (%)			0.001
Female	45 (12.1%)	76 (20.5%)	
Male	140 (37.7%)	110 (29.6%)	
T stage, *n* (%)			0.288
T1	98 (26.6%)	83 (22.6%)	
T2	46 (12.5%)	48 (13%)	
T3	34 (9.2%)	46 (12.5%)	
T4	5 (1.4%)	8 (2.2%)	
N stage, *n* (%)			0.622
N0	127 (49.6%)	125 (48.8%)	
N1	1 (0.4%)	3 (1.2%)	
M stage, *n* (%)			1.000
M0	131 (48.5%)	135 (50%)	
M1	2 (0.7%)	2 (0.7%)	
Pathologic stage, *n* (%)			0.269
Stage I	92 (26.5%)	79 (22.8%)	
Stage II	43 (12.4%)	43 (12.4%)	
Stage III	35 (10.1%)	50 (14.4%)	
Stage IV	3 (0.9%)	2 (0.6%)	
Histologic grade, *n* (%)			0.016
G1	35 (9.6%)	20 (5.5%)	
G2	94 (25.7%)	83 (22.7%)	
G3	48 (13.1%)	74 (20.2%)	
G4	6 (1.6%)	6 (1.6%)	
Residual tumor, *n* (%)			0.320
R0	164 (48%)	160 (46.8%)	
R1	6 (1.8%)	11 (3.2%)	
R2	1 (0.3%)	0 (0%)	
Child–Pugh grade, *n* (%)			0.427
A	114 (47.7%)	103 (43.1%)	
B	13 (5.4%)	8 (3.3%)	
C	0 (0%)	1 (0.4%)	
Adjacent hepatic tissue inflammation, *n* (%)			0.486
None	56 (23.9%)	61 (26.1%)	
Mild	53 (22.6%)	46 (19.7%)	
Severe	11 (4.7%)	7 (3%)	
Vascular invasion, *n* (%)			0.104
No	112 (35.6%)	94 (29.8%)	
Yes	48 (15.2%)	61 (19.4%)	
Fibrosis Ishak score, *n* (%)			0.395
0	41 (19.3%)	33 (15.6%)	
½	21 (9.9%)	10 (4.7%)	
¾	13 (6.1%)	15 (7.1%)	
5/6	42 (19.8%)	37 (17.5%)	
Age, median (IQR)	63 (54, 69)	60 (51, 68)	0.062
AFP (ng/ml), median (IQR)	9 (3, 54)	35 (5.5, 1,795.5)	<0.001
Albumin (g/dl), median (IQR)	4 (3.5, 4.3)	4 (3.5, 4.3)	0.715

**Table 2:** BAIAP2L2 expression associated with clinicopathologic characteristics (logistic regression).

Characteristics	Total (N)	Odds Ratio (OR)	*p*–value
Gender (Female vs. Male)	371	2.149 (1.382–3.372)	<0.001
Age (>60 vs. ≤60 years)	370	0.662 (0.438–0.996)	0.048
T stage (T3 / T4 vs. T1 / T2)	368	1.522 (0.949–2.459)	0.083
N stage (N1 vs. N0)	256	3.048 (0.384–62.072)	0.337
M stage (M1 vs. M0)	270	0.970 (0.115–8.184)	0.976
Pathologic stage (Stage III / Stage IV vs. Stage I / Stage II)	347	1.514 (0.935–2.470)	0.093
Histologic grade (G3 / G4 vs. G1 / G2)	366	1.855 (1.208–2.867)	0.005
Residual tumor (R2 / R1 vs. R0)	342	1.611 (0.619–4.474)	0.337
Child–Pugh grade (B / C vs. A)	239	0.766 (0.305–1.851)	0.558
Adjacent hepatic tissue inflammation (Mild / Severe vs. None)	234	0.760 (0.454–1.270)	0.296
Vascular invasion (Yes vs. No)	315	1.514 (0.951–2.423)	0.082
Fibrosis Ishak score (3/4 / 5/6 vs. 0 / 1/2)	212	1.363 (0.793–2.353)	0.264
AFP (ng/ml) (>400 vs. ≤400)	278	2.544 (1.441–4.586)	0.002
Albumin (g/dl) (≥3.5 vs. <3.5)	297	0.965 (0.562–1.658)	0.896

**Figure 6:**
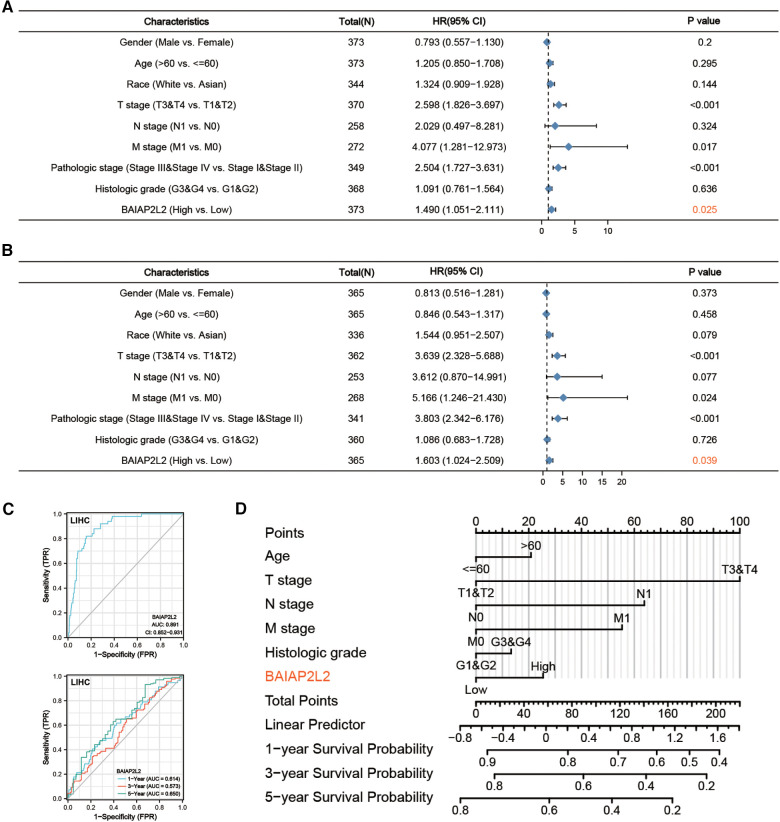
Forest plot, ROC curve and nomogram. (**A**) Forest plot of the Cox regression analysis in TCGA-LIHC (OS). (**B**) Forest plot of the Cox regression analysis in TCGA-LIHC (RFS). (**C**) ROC curve and time-dependent ROC curve for BAIAP2L2 in LIHC samples and adjacent Normal tissue samples from TCGA. (**D**) A nomogram for predicting the 1-, 3- and 5-year survival probability of patients.

Additionally, a receiver operating characteristic (ROC) curve was carried out to fully evaluate the diagnostic value of BAIAP2L2 for LIHC. The area under the curve (AUC) of BAIAP2L2 was 0.891, which suggested high diagnostic value (Figure 6C). The time-dependent ROC curve demonstrated that BAIAP2L2 could accurately predict prognosis (Figure 6C). Moreover, univariate Cox analysis showed that high BAIAP2L2 expression was dramatically correlated with poor OS [hazard ratio (HR) = 1.490, 95% CI =1.051–2.111, *p *= 0.025] and DFS (HR = 1.603, 95% CI = 1.024–2.509, *p *= 0.039) (Figures 6A,B). Eventually, a survival prediction nomogram using age, T stage, N stage, M stage, histologic grade and BAIAP2L2 was used to predict the 1-, 3-, and 5-year survival probability in LIHC (Figure 6D).

### BAIAP2L2 expression-correlated genes and proteins in LIHC

**Figure 7:**
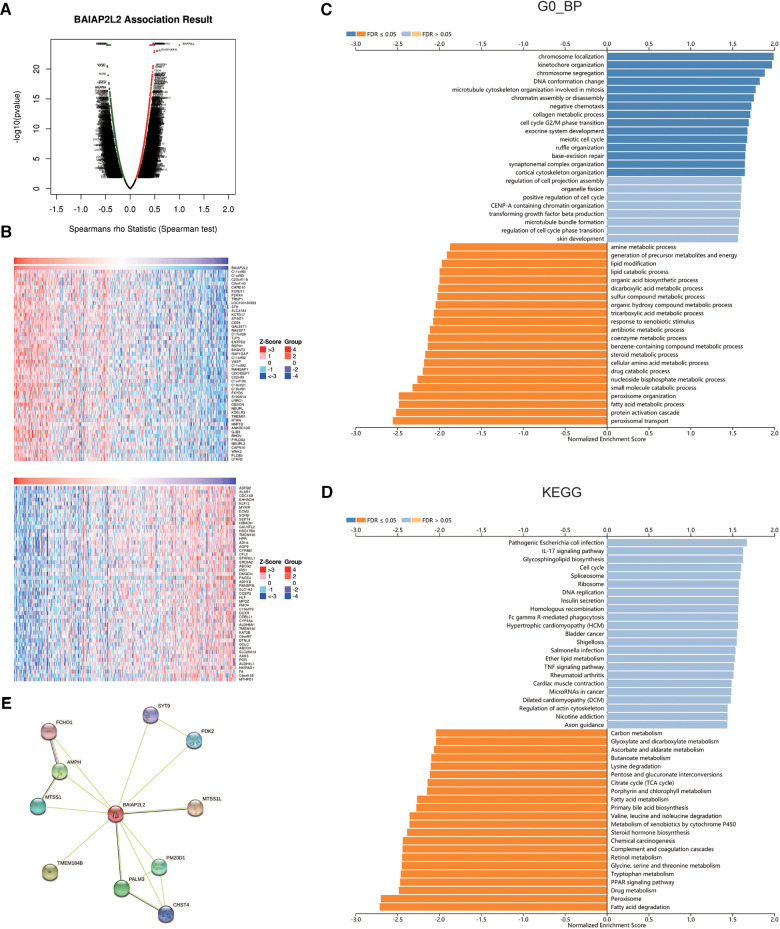
Genes and proteins coexpressed with BAIAP2L2 in LIHC from the LinkedOmics database. (**A**) Highly correlated genes identified by the Pearson test in the LIHC cohort. (**B**) Heat maps showing the top 50 genes positively and negatively correlated with BAIAP2L2 in LIHC (red: positively correlated genes; blue: negatively correlated genes). (**C,D**): Significantly enriched GO annotations and KEGG pathways of the genes coexpressed with BAIAP2L2 in LIHC. (**E**) A PPI network of BAIAP2L2 was generated using the STRING database.

To further investigate the molecular mechanism of BAIAP2L2 in tumorigenesis, we attempted to screen out BAIAP2L2 expression-correlated genes and BAIAP2L2-binding proteins for a series of pathway enrichment analyses. The coexpression network of BAIAP2L2 was constructed by the LinkedOmics database. Figure 7A revealed the genes associated with BAIAP2L2 expression in the LIHC cohort. The 50 genes with the strongest positive and negative correlations are shown in Figure 7B. Then, GSEA was used to analyze the GO and KEGG enrichment analysis of genes coexpressed with BAIAP2L2. GO analysis revealed that genes coexpressed with BAIAP2L2 were mainly involved in chromosome localization and amine kinetochore organization (Figure 7C). KEGG pathway analysis showed that coexpressed genes were involved in fatty acid degradation and peroxisomes (Figure 7D). A PPI network of BAIAP2L2 was established (Figure 7E), showing that BAIAP2L2 interacts with MTSS1, AMPH, FCHO1, SYT9, PDK2, MTSS1L, PM20D1, CHST4 and PALM3. It has been reported that MTSS1 is a novel biomarker of tumor and elevated MTSS1 expression is associated with poor prognosis of liver cancer (40, 41).

### Immune infiltration analysis

**Figure 8:**
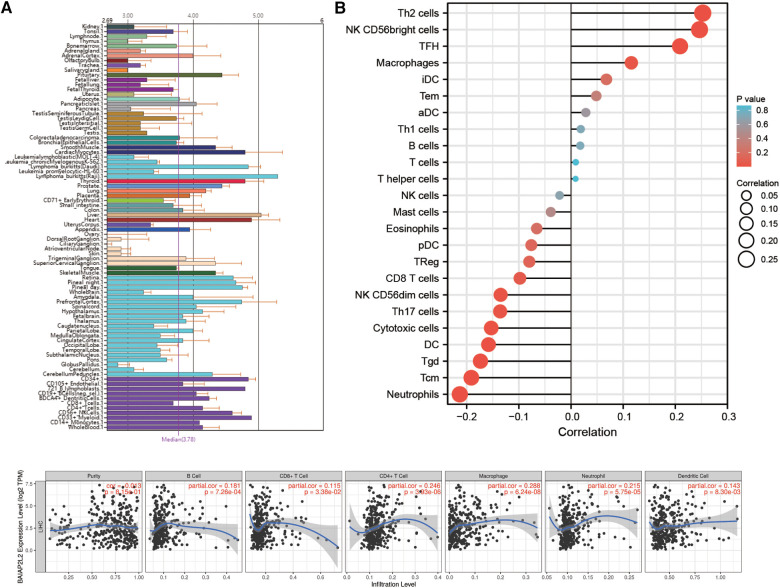
Correlation of BAIAP2L2 expression with the immune infiltration level. (**A**) Immune cell infiltration analysis of BAIAP2L2 in BioGPS. (**B**) The forest plot shows the correlation between BAIAP2L2 expression level and 24 immune cells. (**C**) BAIAP2L2 is significantly associated with tumor purity and is positively correlated with the infiltration of different immune cells using the TIMER database.

Immune cells within the tumor microenvironment (TME) play important roles in tumorigenesis (42, 43). We used ssGSEA, TIMER, BioGPS and GEPIA to investigate the potential relationship between the infiltration level of different immune cells and BAIAP2L2 gene expression in LIHC. First, as shown in the BioGPS results in Figure 8A, higher expression of BAIAP2L2 was observed in B cells, dendritic cells (DCs), CD8+ T cells, CD4+ T cells, natural killer (NK) cells and monocytes. Meanwhile, we also observed that BAIAP2L2 was markedly overexpressed in liver tissue (Figure 8A). Then, we explored the association between BAIAP2L2 and the immune cell infiltration level quantified by ssGSEA in LIHC using Spearman correlation. The results showed that high BAIAP2L2 expression was positively correlated with the infiltration levels of T cells and NK cells (Figure 8B). The TIMER database further showed that the expression of BAIAP2L2 in LIHC was positively correlated with tumor infiltrating cells, including B cells, CD8+ T cells, CD4+ T cells, macrophages, and DCs (Figure 8C). Moreover, in addition to the correlation between BAIAP2L2 and the above immune infiltrating cells, we next sought to determine whether BAIAP2L2 was associated with the expression of more immune infiltrating cells by investigating related immune cell markers in LIHC in TIMER and GEPIA. The results showed that these immune cell markers were related to liver cancer, including B cells, CD8+ T cells, T follicular helper (Tfh) cells, T cells (general), Th1, Th2, Th9, Th17, Th22, Treg, exhausted T cells, M1 and M2 macrophages, tumor-associated macrophages (TAMs), monocytes, NK cells, neutrophils, and DCs (Tables 3, 4).

**Table 3:** Correlation analysis between BAIAP2L2 and gene markers of immune cells in TIMER (22).

Cell type	Gene marker	LIHC (*n *= 371)			
		None		Purity	
		cor	*p*	cor	*p*
B cell	CD19	0.115	*	0.117	*
	CD21 (CR2)	0.238	****	0.257	****
	CD22	0.199	***	0.18	***
T cell (general)	CD3D	0.22	****	0.24	****
	CD3E	0.112	*	0.133	*
	CD2	0.134	**	0.153	**
Th1	STAT4	0.181	***	0.176	**
	STAT1	0.117	*	0.125	*
	CD94 (KLRD1)	−0.115	*	−0.117	*
	IL12RB2	−0.164	**	−0.164	**
	IL27RA	0.324	****	0.329	****
	TNF	0.134	**	0.154	**
Th2	GATA3	0.135	**	0.141	**
	CD184 (CXCR4)	0.184	***	0.182	***
Th9	TGFBR2	−0.139	**	−0.157	**
	IRF4	0.108	*	0.116	*
	SPI1	0.196	***	0.227	****
	TNF	0.134	**	0.154	**
Th17	IL21R	0.134	**	0.144	**
Th22	CCR10	0.237	****	0.223	****
Treg	IL2RA	0.138	**	0.156	**
	CCR8	0.127	*	0.142	**
	TGFB1	0.332	****	0.324	****
Exhausted T cell	PD-1 (PDCD1)	0.173	***	0.182	***
	TIM-3 (HAVCR2)	0.192	***	0.228	****
	CTLA4	0.172	***	0.186	***
	LAG3	0.146	**	0.136	*
M1 Macrophage	IRF5	0.299	****	0.308	****
	COX2 (PTGS2)	0.141	**	0.146	**
M2 Macrophage	ARG1	−0.22	****	−0.205	***
	MRC1	−0.157	**	−0.159	**
TAMs	CD80	0.125	*	0.151	**
	IL10	0.095	0.0665	0.106	*
	CD68	0.138	**	0.147	**
Monocyte	CD86	0.133	*	0.15	**
	CD14	−0.193	***	−0.175	**
NK cell	NCAM1	0.177	***	0.193	***
	CD94 (KLRD1)	−0.115	*	−0.117	*
	CD7	0.223	****	0.235	****
Neutrophil	CD66b (CEACAM8)	0.155	**	0.176	**
	CD11b (ITGAM)	0.159	**	0.166	**
	CD15 (FUT4)	0.389	****	0.373	****
DCs	ITGAX	0.198	***	0.229	****

* *p* < 0.05, **p < 0.01, ***p < 0.001, ****p < 0.0001.

**Table 4:** Correlation analysis between BAIAP2L2 and gene markers of immune cells in GEPIA (31).

Cell type	Gene marker	LIHC			
		Tumor		Normal	
		R	*p*	R	*p*
B cell	CD19	0.15	**	0.38	**
	CD20 (MS4A1)	0.027	0.6	0.44	**
	CD21 (CR2)	0.25	****	0.31	*
	CD22	0.22	****	0.011	0.94
	CD23	−0.076	0.14	0.4	**
	CD24	0.5	****	0.71	****
	CD40	0.024	0.64	0.32	*
	CD72	−0.004	0.94	0.39	**
	CD79a	0.053	0.31	0.55	****
	CD138	−0.0048	0.93	0.36	*
CD8+ T cell	CD8A	0.02	0.7	0.62	****
	CD8B	0.02	0.7	0.61	****
Tfh	CXCR3	0.13	**	0.55	****
	CXCR5	0.25	****	0.2	0.16
	ICOS	0.11	*	0.54	****
T cell (general)	CD3D	0.2	***	0.56	****
	CD3E	0.1	*	0.56	****
	CD2	0.12	*	0.52	***
Th1	IFN-*γ* (IFNG)	0.02	0.7	0.38	**
	STAT4	0.18	***	0.41	**
	STAT1	0.15	**	0.53	****
	CD94 (KLRD1)	−0.077	0.14	0.33	*
	IL12RB2	−0.1	*	0.34	*
	IL27RA	0.33	****	0.44	**
Th2	GATA3	0.12	*	0.27	0.063
	STAT6	0.14	**	0.41	**
	CD184 (CXCR4)	0.2	***	0.57	****
	CD194 (CCR4)	0.14	**	0.41	**
Th9	SPI1	0.21	****	0.39	**
	TNF	0.14	**	0.35	*
Th17	IL21R	0.13	*	0.47	***
	IL23R	0.036	0.49	0.33	*
	CD161 (KLRB1)	0.042	0.42	0.29	*
Th22	CCR10	0.22	****	0.28	*
Treg	IL2RA	0.15	**	0.44	**
	FOXP3	−0.0077	0.88	0.38	**
	CCR8	0.17	**	0.23	0.12
	CD127 (IL7R)	0.1	*	0.55	****
	TGFB1	0.33	****	0.5	***
Exhausted T cell	PD-1 (PDCD1)	0.16	**	0.67	****
	TIM-3 (HAVCR2)	0.19	***	0.31	*
	CTLA4	0.14	**	0.59	****
	LAG3	0.12	*	0.19	0.2
M1 Macrophage	IRF5	0.33	****	0.27	0.057
	COX2 (PTGS2)	0.15	**	0.31	*
	INOS (NOS2)	−0.019	0.71	0.46	***
M2 Macrophage	ARG1	−0.18	***	0.005	0.97
	MRC1	−0.13	*	0.086	0.55
	VSIG4	0.064	0.22	0.29	*
	MS4A4A	−0.011	0.84	0.32	*
TAMs	CD80	0.13	*	0.34	*
	IL10	0.074	0.16	0.35	*
	CD68	0.14	**	0.32	*
Monocyte	CD86	0.14	**	0.36	*
	CD14	−0.17	**	−0.16	0.28
NK cell	NCAM1	0.16	**	0.38	**
	CD94 (KLRD1)	−0.077	0.14	0.33	0.02
	CD7	0.19	***	0.37	**
Neutrophil	CD66b (CEACAM8)	0.15	**	0.47	***
	CD11b (ITGAM)	0.16	**	0.47	***
	CD15 (FUT4)	0.41	****	0.55	****
	CCR7	0.027	0.61	0.42	**
	MPO	−0.055	0.29	0.5	***
DCs	CD1C	0.11	*	0.21	0.14
	CD141	0.027	0.6	0.42	**
	HLA-DPB1	0.07	0.18	0.38	**
	HLA-DRA	0.035	0.5	0.4	**
	THBD	0.027	0.6	0.42	**
	NRP1	0.089	0.089	0.29	*
	ITGAX	0.21	****	0.41	**

* *p* < 0.05, **p < 0.01, ***p < 0.001, ****p < 0.0001.

## Discussion

In recent years, studies have suggested that BAIAP2L2 may be involved in the development of human cancer (36, 37, 44). However, the relationship between BAIAP2L2 and liver cancer has not been reported. Hence, we performed a comprehensive bioinformatics analysis of BAIAP2L2 expression and survival prognostic value in LIHC. Our results reveal for the first time that Overexpression of BAIAP2L2 is associated with poor prognosis of LIHC.

Our results showed that BAIAP2L2 was upregulated in BLCA, CHOL, ESCA, HNSC, KIRC, LIHC, LUAD, LUSC, PAAD, PRAD and STAD based on TCGA. In 2020, Liu et al. found that BAIAP2L2 is highly expressed in STAD, and it can promote proliferation, migration and invasion and ultimately induce apoptosis of gastric cancer cells (20). BAIAP2L2 was also upregulated in PRAD, and it may promote tumorigenesis and malignant development (37). Upregulation of BAIAP2L2 was detected in various lung cancer cell lines and was deemed a novel biomarker and potential therapeutic target for LUAD (22). These reports support the results of this study. However, the relationship between BAIAP2L2 and LIHC has not been reported. Moreover, we found that BAIAP2L2 was significantly overexpressed in LIHC (*p* < 0.001). Interestingly, in combination with the survival analysis of the Kaplan–Meier Plotter and LinkedOmics databases, differences in both OS and DFS between the normal and tumor groups were observed only in LIHC. These results imply that BAIAP2L2 may play a unique and crucial role in LIHC.

Subsequently, we validated the expression of BAIAP2L2 mRNA and protein in LIHC using various online databases. All the results showed that BAIAP2L2 expression was upregulated in LIHC. To verify the above results, we performed qRT–PCR on HEPG2 cell and LO2 cell, and the results showed that the expression of BAIAP2L2 in HEPG2 cell was higher than that in LO2 cell.

LIHC is the fourth most common fatal malignancy and the sixth most common in terms of incidence cases in the world (3). The most common primary liver cancer, usually occurs in the context of chronic liver disease and is often diagnosed with liver cancer in advanced stages, resulting in its poor prognosis (45). It is of great significance to explore the pathogenesis of LIHC and identify potential molecular biomarkers. Therefore, we focused on the clinical significance and possible molecular mechanism of BAIAP2L2 in LIHC.

Epigenetic changes have become an emerging applications for cancer biomarkers (46, 47). DNA methylation plays an important role in the development of cancer (48). Thus, we investigated whether the abnormal expression of BAIAP2L2 in cancer is related to epigenetics. According to Figure 3, we can see that high BAIAP2L2 expression was accompanied by gene alterations in LIHC. Furthermore, high BAIAP2L2 levels were associated with lower DNA methylation levels in LIHC. Results suggested that abnormally increased expression of BAIAP2L2 mRNA in LIHC may be the result of both genetic alterations and lower DNA methylation levels.

Additionally, we found that a high level of BAIAP2L2 indicated unfavorable survival probability in LIHC. Logistic regression analysis showed that high BAIAP2L2 expression was correlated with sex, age, histologic grade and alpha fetoprotein (AFP). It is well known that AFP is the most widely used serum biomarker for the diagnosis of primary liver cancer worldwide and is associated with poor prognosis (49). Simultaneously, Cox regression revealed that upregulation of BAIAP2L2 was an independent prognostic factor for poor prognosis of LIHC, along with pathological stage, T stage and distant metastasis (Figure 6). ROC analysis also indicated that BAIAP2L2 had a high diagnostic value in LIHC, and its AUC was 0.89. More importantly, a prognostic nomogram including age, T, M, N typing, histologic grade and BAIAP2L2 was constructed. The nomogram results suggested that BAIAP2L2 can reflect the prognosis of LIHC to some extent. These results demonstate that BAIAP2L2 plays an important role in the development of LIHC and may be an independent prognostic biomarker of LIHC.

To further investigate the molecular mechanism of the BAIAP2L2 gene in tumorigenesis, GSEA was used to analyze the GO and KEGG enrichment analysis of genes coexpressed with BAIAP2L2. The results suggest that MTSS1 has synergistic effect with BAIAP2L2. Huang et al. found that elevated MTSS1 expression is associated with poor prognosis of LIHC (41). In other words, MTSS1 and BAIAP2L2 may play a synergistic role in the carcinogenesis of LIHC.

In recent years, increasing evidence has shown that immune infiltration is closely related to malignant tumors (50–54). Therefore, we further analyzed the relationship between the carcinogenic effect of BAIAP2L2 and immune infiltration. Higher expression of BAIAP2L2 was observed in B cells, DCs, CD8+ T cells, CD4+ T cells, natural killer (NK) cells and monocytes in the BioGPS. Concurrently, the TIMER database showed that the expression of BAIAP2L2 in LIHC was positively correlated with tumor infiltrating cells, including B cells, DCs, CD8+ T cells, CD4+ T cells and macrophages. Moreover, LIHC was associated with immune cell markers, including B cells, CD8+ T cells, Tfh cells, T cells (general), Th1, Th2, Th9, Th17, Th22, Treg, exhausted T cells, M1 and M2 macrophages, TAMs, monocytes, NK cells, neutrophils, and DCs. Single-cell sequencing showed that CD8+ T cells were associated with liver cancer (55). Clinical samples also showed that immune cell markers were related to liver cancer, including B cells, Tfh cells, M1 macrophages, NK cells and neutrophils (56). Our findings are consistent with both studies. In summary, immune infiltration plays a crucial role in carcinogenesis.

Nevertheless, although we employed multiple bioinformatics databases to analyze the role of BAIAP2L2 in LIHC, this study still has some limitations. Firstly, although bioinformatics analysis is a powerful and efficient tool to help understand the molecular mechanisms and to identify potential biomarkers of LIHC, further experimental validations, such as evidence obtained from western blot and immunohistochemistry assays, are needed to confirm the prognosis value and immunological role of BAIAP2L2 in LIHC. Secondly, because most of the data come from public databases, there may be some biases caused by potential confounding factors. Finally, It appears that a single biomarker would lack enough prognosis power. Multiple biomarkers should be included to build a prognosis model to improve prognosis value. Unable to incorporate more hub genes is one of the limitations of our study. In future studies, we will try to combine hub gene to build a new prognosis model to improve specifificity and we will further validated in cell lines and animal models.

In conclusion, this is the first study to demonstrate the high expression of BAIAP2L2 and its prognostic value in LIHC. Our results also hinted at the potential role of BAIAP2L2 in modulating immune infiltration. These data provide a reference for future understanding of the role of BAIAP2L2 in LIHC.

## Conclusion

In summary, a pan-cancer analysis shows that BAIAP2L2 is highly expressed in LIHC and overexpression of BAIAP2L2 is associated with poor prognosis of LIHC. Furthermore, BAIAP2L2 may be an independent prognostic biomarker of LIHC and be associated with immune infiltration. Nevertheless, the specific role and precise regulatory mechanism of BAIAP2L2 in LIHC need further far-ranging and thorough research.

## Data Availability

The datasets presented in this study can be found in online repositories. The names of the repository/repositories and accession number(s) can be found below: The data can be accessed by following websites: https://cistrome.shinyapps.io/timer/, http://starbase.sysu.edu.cn/, http://ualcan.path.uab.edu/analysis.html, https://www.proteinatlas.org/, https://kmplot.com/analysis/, http://www.linkedomics.org/, https://string-db.org/, http://biogps.org/.
